# *OsLUX* Confers Rice Cold Tolerance as a Positive Regulatory Factor

**DOI:** 10.3390/ijms24076727

**Published:** 2023-04-04

**Authors:** Peng Huang, Zhengquan Ding, Min Duan, Yi Xiong, Xinxin Li, Xi Yuan, Ji Huang

**Affiliations:** 1 College of Life Sciences, Zhejiang Normal University, Jinhua 321004, China; 2 Jiaxing Academy of Agricultural Sciences, Jiaxing 314016, China; 3 Taizhou Academy Agricultural of Sciences, Taizhou 317000, China; 4 State Key Laboratory of Crop Genetics and Germplasm Enhancement, College of Agriculture, Nanjing Agricultural University, Nanjing 210095, China

**Keywords:** rice, *LUX ARRHYTHMO*, cold stress, DREB, transcriptional regulation

## Abstract

During the early seedling stage, rice (*Oryza sativa* L.) must overcome low-temperature stress. While a few cold-tolerance genes have been characterized, further excavation of cold-resistance genes is still needed. In this study, we identified a cold-induced transcription factor—LUX ARRHYTHMO (LUX)—in rice. OsLUX was found to be specifically expressed in leaf blades and upregulated by both cold stress and circadian rhythm. The full-length OsLUX showed autoactivation activity, and the OsLUX protein localized throughout the entire onion cell. Overexpressing *OsLUX* resulted in increased cold tolerance and reduced ion leakage under cold-stress conditions during the seedling stage. In contrast, the knockout of *OsLUX* decreased seedling cold tolerance and showed higher ion leakage compared to the wild type. Furthermore, overexpressing *OsLUX* upregulated the expression levels of oxidative stress-responsive genes, which improved reactive oxygen species (ROS) scavenging ability and enhanced tolerance to chilling stress. Promoter analysis showed that the *OsLUX* promoter contains two dehydration-responsive element binding (DREB) motifs at positions −510/−505 (GTCGGa) and −162/−170 (cCACCGccc), which indicated that OsDREB1s and OsDREB2s probably regulate *OsLUX* expression by binding to the motif to respond to cold stress. Thus, OsLUX may act as a downstream gene of the DREB pathway. These results demonstrate that OsLUX serves as a positive regulatory factor of cold stress and that overexpressing OsLUX could be used in rice breeding programs to enhance abiotic stress tolerance.

## 1. Introduction

Rice (*Oryza sativa* L.) is an important staple food worldwide, especially in Asia. However, cold stress is a major abiotic stress that can adversely affect plant growth and development defects, and reduced crop yields [1,2,3,4]. Under low-temperature stress, rice seedlings grow slowly, leaves turn yellow or even wither, and plants grow stunted or even die. To maintain normal development under cold stress, plants have gradually evolved a response mechanism that specifically responds to stress, in which the expression regulation of stress-related genes is a key regulatory mechanism [5,6,7]. Therefore, it is particularly important to further explore cold-tolerant transcription factors to prevent low-temperature damage to rice.

Transcription factors are critical proteins involved in regulating gene expression at the transcriptional level under cold stress [8,9]. The DREB1/CBF (DRE-binding/C-repeat binding factor) protein belongs to the APETALA2 (AP2) family of transcription factors and is considered to be one of the key components of cold acclimatization [10,11,12,13]. The functions of DREB1/CBFs orthologous genes in rice, namely *OsDREB1A*, *OsDREB1B*, and *OsDREB1C*, have been identified to regulate cold tolerance [13,14,15,16,17]. The MYB transcription factor family is one of the most abundant transcription factor families in plants, and a large number of MYB transcription factors have been isolated and identified in plants [3,18]. Among them, the functions of a few MYB family members were found to regulate cold tolerance in rice. OsMYB3R-2 could be induced by cold stress, specifically bind to the *cis*-element of the mitosis-specific activator in the *OsCycB1;1* promoter, activating its expression to regulate cell cycle progression under cold conditions, thereby enhancing cold tolerance in rice [19,20]. Moreover, the expression of genes related to the DREB1/CBF pathway in *OsMYB3R-2*-overexpression transgenic plants was significantly higher than that in wild-type plants, so OsMYB3R-2 may coordinate the cell cycle and DREB/CBF pathway to improve the cold tolerance of rice [20]. Overexpression of *OsMYB2* improved the survival rate under cold stress, as OsMYB2 could also activate the expression of DREB1/CBF pathway-related genes [21].

The expression of OsMYBS3 was also induced by low temperature. Compared to wild-type and transgenic RNAi rice, overexpression of OsMYBS3 exhibited more cold tolerance and could regulate the expression of multiple cold-tolerance genes (*TPP1*, *TPP2*, *WRKY77*) to enhance the plant’s ability to adapt to cold stress and protect plant cells from cold damage. Further studies have shown that OsMYBS3 can inhibit the expression of DREB1 family genes and their downstream genes under cold stress, thereby participating in the regulation of the DREB/CBF pathway, which responds rapidly to cold stress. DREB1 and MYBS3 provided two complementary mechanisms for cold tolerance in rice, with DREB1 mediating the short-term cold-stress response and MYBS3 regulating the long-term adaptation of rice to cold stress [3,22]. Interestingly, the overexpression of OsMYB30 in rice increased the sensitivity to chilling stress, while the knockout mutant of the OsMYB30 gene increased the tolerance to chilling stress. Further analysis showed that OsMYB30 could interact with the jasmonic acid signal response pathway-related protein OsJAZ9 and directly bind to the OsJAZ9 promoters to repress the gene expression of β-amylase (BMY), and the maltose produced by starch decomposition could protect cells under low-temperature stress. Thus, OsMYB30 is a negative regulator of cold tolerance, which can interact with the OsJAZ9 protein to negatively regulate the BMY gene, thereby regulating the content of starch and maltose and controlling the response of plants to cold stress [23,24].

The circadian rhythm is an internal biological clock that enables plants to synchronize their physiological and developmental processes with the 24 h light/dark cycle [25,26]. The EC (evening complex) is an important component in photoperiod flowering in *Arabidopsis*, composed of LUX ARRYTHMO (LUX), EARLY FLOWERING 3 (ELF3) and EARLY FLOWERING 4 (ELF4) [27,28]. The *LUX ARRYHTHMO (LUX)* gene, encoding a MYB family transcription factor protein, is a key component of the circadian clock in *Arabidopsis* [28,29,30,31,32]. *OsLUX* (also known as *OsPCL1*), the Arabidopsis *LUX* orthologous gene in rice, also plays a critical role in regulating rice heading [32,33,34,35,36]. OsLUX can form the OsEC (OsELF4s–OsELF3-1–OsLUX) complex to bind to the promoters of *Hd1* and *Ghd7*, which suppress *Hd1* and *Ghd7* expression to regulate heading in rice [34,35]. Additionally, the genes involved in circadian rhythms play important roles in plant stress responses [37,38]. Abiotic stresses such as drought, high salinity, and extreme temperatures can disrupt the plant’s circadian rhythm and negatively impact its growth and survival [39,40]. The bridge has been established to link the cold stress and circadian rhythms in *Arabidopsis* [41,42,43]. The loss-of-function mutations of clock proteins CIRCADIAN CLOCK-ASSOCIATED 1 (CCA1)/LATE ELONGATED HYPOCOTYL (LHY) showed more sensitivity to cold stress, whereas CCA1 overexpression enhanced the tolerance to cold stress [41,42,44]. Recent research on the *LUX* has investigated the relationship between circadian rhythms and abiotic stress tolerance in *Arabidopsis* and rice [35,43]. It has shown that CBF1/DREB1b binds to the CRT in the *LUX* promoter to regulate the *LUX* transcriptional expression involved in the regulation of cold-stress responses in *Arabidopsis*. However, the coordinated regulatory networks between circadian rhythms and cold tolerance in rice are still largely unknown.

In this study, we discovered that the transcription factor OsLUX exhibited a rhythmic pattern and is induced by cold stress in rice. Overexpression of *OsLUX* enhances cold tolerance in seedlings, while the knockout of *OsLUX* reduces seedling cold tolerance and increases ion leakage. Furthermore, overexpression of *OsLUX* improves ROS scavenging ability and enhances tolerance to chilling stress by upregulating the expression levels of *OsAPX1*, *OsAPX2*, and *OsPOX1*. The *OsLUX* promoter contains two DREB-binding elements motifs at positions −510/−505 (GTCGGa) and −162/−170 (cCACCGccc), which indicates that the OsDREB1s and OsDREB2s probably regulate OsLUX expression. Taken together, our findings demonstrate that OsLUX serves as a positive regulatory factor of cold stress and plays a crucial role in integrating cold-stress responses and circadian rhythms in rice. These results suggest that LUX plays an essential role in regulating the plant’s circadian rhythm and integrating it with abiotic stress signaling pathways, which could have significant implications for crop breeding and agricultural productivity in the face of climate change.

## 2. Results

### 2.1. Expression Pattern of the OsLUX Gene

**Figure 1:**
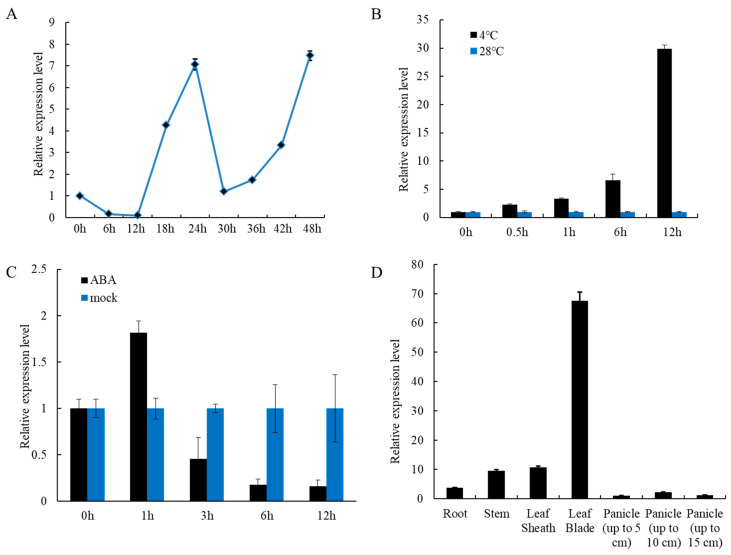
Expression pattern analysis of *OsLUX* in rice seedlings. (**A**) The expression of *OsLUX* response to circadian rhythm was carried out within 48 h, and the expression level was detected every 6 h during this period. (**B**) The expression levels of *OsLUX* were compared after low-temperature (4 °C) treatment and normal conditions (28 °C) at 0 h, half an hour, 1 h, 6 h and 12 h. (**C**) The expression level of *OsLUX* with ABA treatment (100 μM) after 0 h, 1 h, 3 h, 6 h and 12 h. Mock represents the treatment with H_2_O control. (**D**) Tissue-specific gene expression analysis of *OsLUX* at booting stage. The data are the means and SD of three replicates.

To understand the expression pattern of the *OsLUX* gene, we examined it through reverse-transcription quantitative PCR (RT-qPCR) analysis in different organs of 14-week-old wild-type rice (*O. sativa* L. *japonica*. cv. Zhonghua11, ZH11) grown in Yoshida’s nutrient solution for hydroponic culture. The results showed that *OsLUX* expression exhibited a rhythmic pattern consistent with previous studies [33,34,35] (Figure 1A Figure S1). We also investigated the response of *OsLUX* expression to cold stress and ABA treatment. The RT-qPCR assay revealed that *OsLUX* expression was induced by cold stress, with transcript levels peaking at 12 h after treatment (Figure 1B). Furthermore, *OsLUX* expression was significantly upregulated after one hour of exogenous ABA treatment, but then rapidly decreased (Figure 1C). Interestingly, the expression pattern of *OsLUX* under cold stress differed from that under ABA treatment. Additionally, the *OsLUX* transcript was found to be widely distributed in all organs, with high expression levels particularly in leaf blades (Figure 1D).

### 2.2. Subcellular Localization of OsLUX Protein 

**Figure 2:**
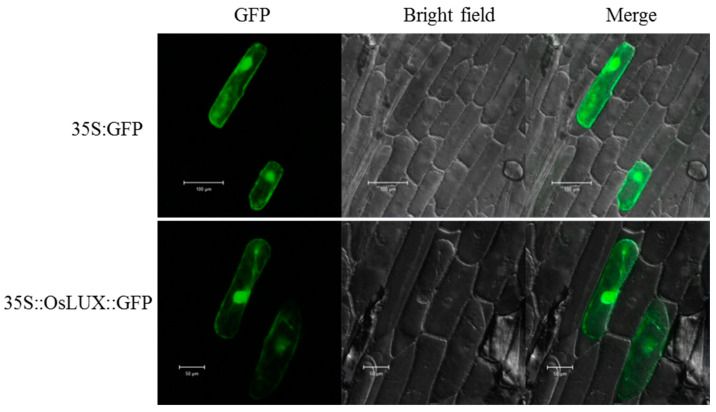
Subcellular localization of OsLUX. The subcellular localization of OsLUX:GFP in onion (*Allium cepa*) epidermal cells by particle bombardment and detected the fluorescence by confocal laser scanning microscopy. (scale bar = 50 μm). The localization was examined under fluorescence and bright field.

To determine the subcellular localization of OsLUX, we cloned the *OsLUX* coding sequence in the pA7-GFP vector fused to the N-terminus of the gene-encoding enhanced green fluorescent protein (GFP) under the control of the CaMV 35S promoter. We then transiently expressed the construct expressing the OsLUX-GFP (OsLUX:GFP) fusion protein or the empty vector pA7-GFP (GFP) in isolated onion (*Allium cepa* L.) epidermal cells by particle bombardment and detected the fluorescence by confocal laser scanning microscopy. In the onion cells expressing OsLUX:GFP, the green fluorescent signals were observed throughout the cell, including the nucleus and the cytoplasm, similar to the green fluorescent signals of GFP alone (Figure 2).

### 2.3. OsLUX Functions as a Transcription Factor

**Figure 3:**
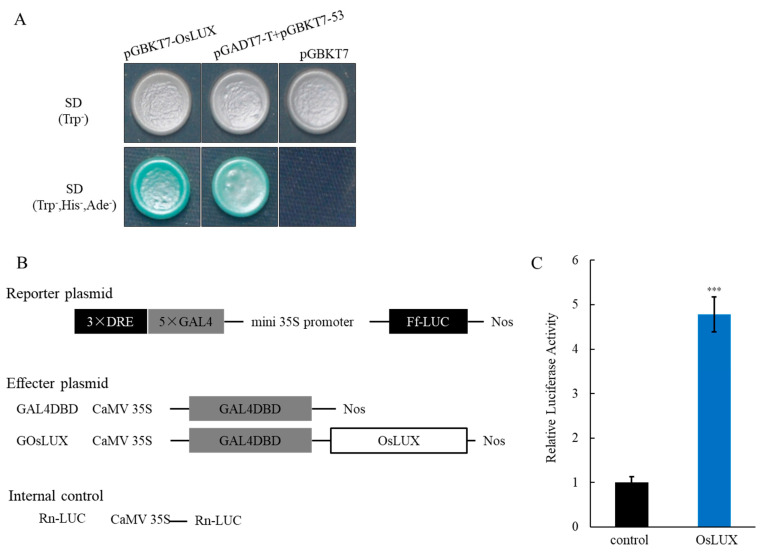
Transcriptional activity analysis of OsLUX. (**A**) A GAL4 DNA-binding domain-OsMYB511-fused protein (pGBKT7-OsLUX) was expressed in AH109 yeast cells growing on SD/Trp- and SD/Trp-/His-/Ade medium, respectively. pGADT7-T and pGBKT7-53 expressed as a positive control, pGBKT7 as a negative control in AH109 yeast cells. (**B**) Schematic diagram of the reporter and effector plasmids used in the rice protoplast transfection assay. The reporter plasmid contains a firefly LUC reporter gene (Ff-LUC), driven by three copies of DREs, five copies of the GAL4 and a mini 35S promoter. The effector containing DREB1A on a reporter plasmid. Other effector plasmids containing the GAL4DBD bound the GAL4 sites. Rn-LUC serves as the internal control. (**C**) Relative LUC activity assay in rice protoplast that had been transfected with both effecter plasmid GOsLUX and reporter plasmids. The effecter plasmid GAL4DBD and reporter plasmid co-transfection used as the control. Data are the means and SD of three replicates, *** *p* < 0.001, Student *t*-test.

To test whether OsLUX has transcription activity, OsLUX was fused in-frame to the GAL4 DNA-binding domain (BD) in the pGBKT7 (pBD) vector and the fusion constructs pBD-OsLUX were transformed into the yeast cell (*Saccharomyces cerevisiae*). As shown in Figure 3A, the transformants containing pBD-OsLUX and the positive control harboring pAD-T + pBD-53 could grow normally on SD/–Trp/– His/–Ade medium exclusively and exhibited the activity of β-galactosidase reporter gene upon the addition of X-α-gal (Figure 3A). On the other hand, we performed reporter-effector transient expression assays in rice protoplasts. Effector constructs carry OsLUX driven by the CaMV 35S promoter as a detection group or do not carry OsLUX as a control. The reporter plasmid contains a firefly luciferase (Ff-LUC) reporter gene driven by the dehydration-response element (DRE), the GAL4 DNA-binding domain (GAL4DBD), and the minimal CaMV 35S promoter. Renilla luciferase (Rn-LUC) was used to normalize transfection efficiency (Figure 3B). Comparing the LUC activity with the control group, transfection with the OsLUX showed higher relative LUC activity, confirming that OsLUX is a transcription factor with autoactivations (Figure 3C). 

### 2.4. Knockout of OsLUX Enhances Tolerance to Cold Stress in Rice

**Figure 4:**
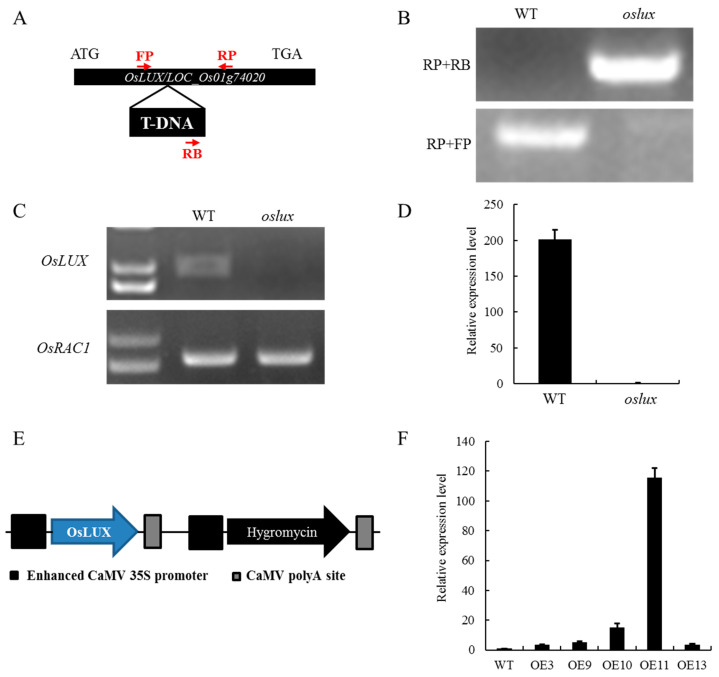
Identification of the *oslux* mutants and *OsLUX*-overexpression plants. (**A**,**B**) Schematic diagram and genotyping of the *oslux* mutants were identified by PCR with gene-specific primers (FP, RP) and T-DNA border primer (BP). (**C**,**D**) Semi-quantitative RT-PCR and real-time quantitative RT-PCR assays to test the transcript levels of the corresponding gene normalized to *OsActin1*. (**E**) To generate overexpression transgenic rice, the coding sequence of *OsLUX* was amplified and cloned into pCAMBIA1300, in which transferred gene expression is under the control of CaMV 35S promoter. (**F**) The T1 transgenic overexpressing plants were selected by RT-qPCR analysis. Data are the means and SD of three replicates.

To investigate the biological function of OsLUX, we obtained a T-DNA insertion mutant line of *OsLUX* in the ZH11 background from the Rice Mutant Database (RMD) in Wuhan, China. We isolated homozygous mutant plants from segregating progeny plants using T-DNA- and gene-specific primers through genomic DNA PCR (Figure 4A,B). We further tested the transcript level of *OsLUX* using RT-PCR and RT-qPCR assays, which showed that the relative expression of *OsLUX* was abolished in homozygous mutant seedlings with the control (Figure 4C,D). In addition, we constructed an overexpression vector under the control of the CaMV 35S promoter and transformed it into wild-type ZH11, resulting in several transgenic lines (Figure 4E). We confirmed the transgenic rice lines through RT-qPCR and found that the *OsLUX* transcript abundance was higher in the *OsLUX*-overexpressing lines (OE3, OE9, OE10, OE11, and OE13) compared to the wild-type rice. We selected three transgenic lines with higher expression levels (OE10, OE11, and OE13) for the subsequent experiments (Figure 4F).

**Figure 5:**
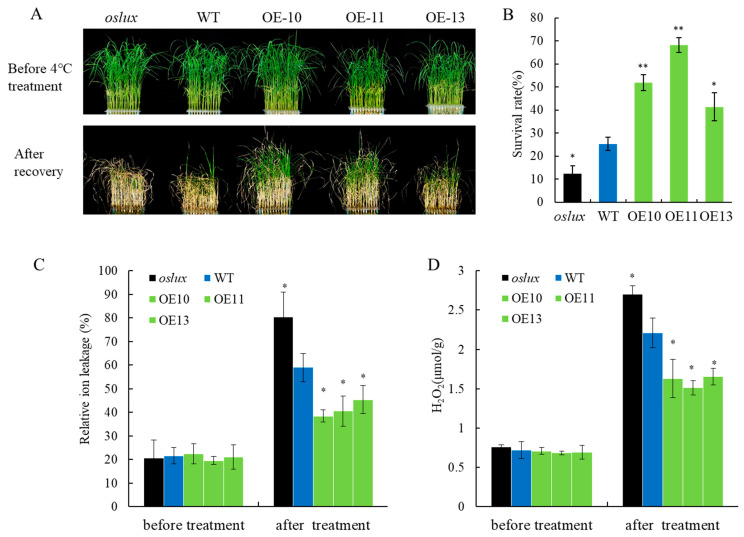
*OsLUX* confers cold tolerance in rice at the seedling stage. (**A**) The phenotyping of *oslux* mutants, *OsLUX*-overexpression plants and wild-type grown to 3 weeks at normal condition and treated with 4 °C for 5 d. The survival rates were counted after re-watering for 7 d. (**B**) The survival rates measured for (**A**). (**C**) The contents of relative ion leakage measured for the (**A**). (**D**) The H_2_O_2_ contents measured for (**A**). Data are the means and SD of three replicates, * *p* < 0.05, ** *p* < 0.01, Student *t*-test.

Under normal non-stressed conditions in a hydroponic solution, there was no difference observed between transgenic and wild-type plants (Figure 5A upper lane). However, following exposure to cold stress (4 °C for 5 d) and subsequent recovery under normal conditions for 7 days, *OsLUX*-overexpressing line seedlings exhibited continuous growth and remained mostly green, whereas the *oslux* mutant seedlings showed severe leaf rolling and wilting compared to the wild-type (Figure 5A bottom lane). The survival rate of *OsLUX*-overexpressing plants was significantly higher than that of wild-type seedlings, while the *oslux* mutant showed a much lower survival rate than wild-type seedlings (Figure 5B). We also measured ion leakage and found that it was significantly increased in the *oslux* mutant after cold treatment, while ion leakage was weaker in *OsLUX*-overexpressing plants than in WT plants (Figure 5C). Abiotic stress can cause an increase in ROS, leading to further damage to plants and even cell death. We observed no significant difference in H_2_O_2_ content between all lines before low-temperature treatment. However, after cold treatment, the H_2_O_2_ content of the *oslux* mutant was significantly higher than that of WT, whereas the H_2_O_2_ content of *OsLUX*-overexpressing plants was significantly lower than that of WT (Figure 5D).

### 2.5. OsLUX-Overexpressing May Activate ROS-Scavenging Ability

**Figure 6:**
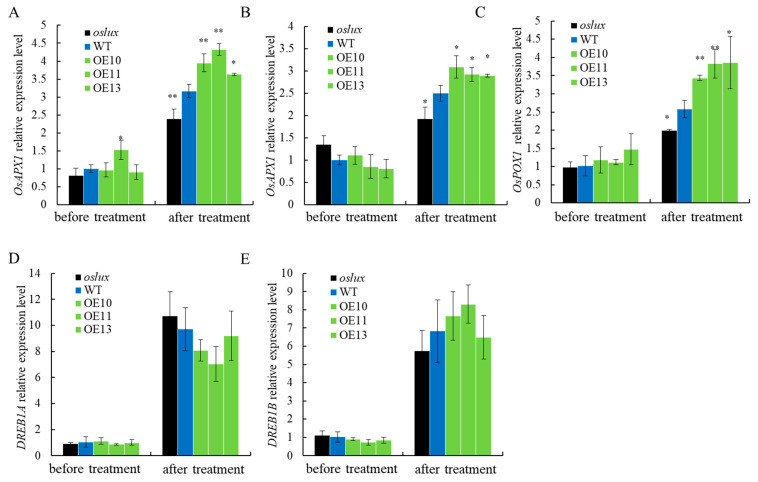
Quantitative real-time PCR analysis of genes associated with ROS scavenging (**A**) *OsAPX1*, (**B**) *OsAPX2*, (**C**) *OsPOX1* and DREB-/CBF-dependent transcriptional regulatory genes (**D**) *DREB1A*, (**E**) *DREB1B*, in *oslux* mutants, *OsLUX*-overexpression plants and wild-type under cold stress and normal condition at seedling stage. Data are the means and SD of three replicates, * *p* < 0.05; ** *p* < 0.01, Student *t*-test.

In order to explore the mechanism of *OsLUX* in regulating plants’ ability to scavenge H_2_O_2_ in rice, we detected the expression of ROS scavenging-related genes [45,46,47,48]. It was discovered that *OsAPX1*, *OsAPX2*, and *OsPOX1* were not significantly changed in all plants before cold treatment. However, after cold treatment, the expression of these genes was significantly upregulated in *OsLUX*-overexpressing lines and generally repressed in *oslux* mutants (Figure 6A–C). The DREB-/CBF-dependent transcriptional regulatory pathway plays an essential role in chilling responses in plants. Therefore, the expression levels of the DREB1A and DREB1B genes were detected. It was found that there was no significant difference in the expression levels of the two genes in all lines before or after low-temperature treatment (Figure 6D,E). The expression of *DREB1A* and *DREB1B* was activated after low-temperature treatment, indicating that they may be independent or upstream of the regulation of OsLUX. Nonetheless, *OsLUX* overexpression improved ROS scavenging ability and enhanced tolerance to chilling stress.

### 2.6. OsLUX May Act as a Downstream Gene of the DREB Pathway

**Figure 7:**
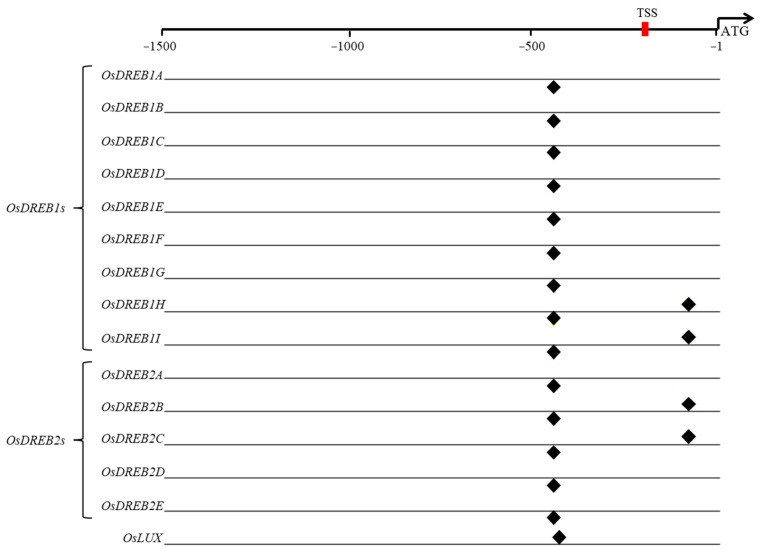
*OsLUX* promoter analysis with PlantPAN3.0. The 1500 base pairs upstream of the *OsLUX* promoter were selected for analysis in PlantPAN3.0. OsDREB1s and OsDREB2s may regulate *OsLUX* expression by binding to the motifs at positions −510/−505 (GTCGGa) and −162/−170 (cCACCGccc) to respond to the cold stress. OsLUX could autoregulate its own expression level at the position −485/−495 (tccGAATCttg). The red box indicates the transcription start site (TSS), and the black diamond indicates the binding position.

We found that there was no significant difference in the expression levels of *DREB1A* and *DREB1B* genes. Therefore, we wondered if OsLUX might be a downstream gene of the DREB/CBF pathway that plays a role in the cold-stress response. To investigate this, we analyzed the 1500 base pairs upstream of the *OsLUX* initiation codon for promoter elements using PlantPAN3.0 [49]. The analysis suggested that the promoter contains two DREB-binding element motifs at positions −510/−505 (GTCGGa) and −162/−170 (cCACCGccc). Both OsDREB1s and OsDREB2s could potentially regulate *OsLUX* expression by binding to the motif at position −510/−505 (GTCGGa) (Figure 7). In addition, *OsDREB1H*, *OsDREB1I*, *OsDREB2B*, and *OsDREB2C* could also bind to the motif at position −162/−170 (cCACCGccc) to regulate *OsLUX* expression in response to cold stress. Furthermore, OsLUX may also autoregulate its own expression level through the motif at position −485/−495 (tccGAATCttg) (Figure 7). Taken together, these findings suggest that OsLUX may be both autoregulated and mediated by OsDREB1s and OsDREB2s as a downstream gene of the DREB/CBF pathway in response to cold stress.

## 3. Discussion

Previous studies have shown that lots of MYB family members regulate rice cold tolerance [3]. In our study, we identified *OsLUX*, a cold-induced transcription factor that encodes an MYB family protein. *OsLUX* expression was highly expressed in leaves and induced by cold treatment (Figure 1B,D). Moreover, transcriptional autoactivation and reporter-effector transient expression assays showed that the OsLUX protein has autoactivation activity, indicating that OsLUX can act as a transcriptional regulator to regulate the expression of downstream target genes in response to cold stress (Figure 3). Furthermore, we obtained knockout and overexpression transgenic lines of *OsLUX* to verify its role in cold stress (Figure 4). The survival rate of the *oslux* mutant was significantly lower than that of wild-type seedlings, whereas the *OsLUX*-overexpressing lines showed much higher survival rates than the wild-type seedlings (Figure 5A,B). The ion leakage and H_2_O_2_ content data showed that the *oslux* mutant exhibited significantly increased levels after cold-stress treatment, while the ion leakage and H_2_O_2_ content of *OsLUX*-overexpressing plants were lower than the WT plants (Figure 5C,D). *OsLUX* overexpression induced the expression of *OsAPX1*, *OsAPX2*, and *OsPOX1*, which improved ROS scavenging ability and enhanced tolerance to chilling stress. Therefore, our results suggest that OsLUX plays a positive regulatory role in cold-stress response in rice.

The DREB1/CBF transcription factor has proved to be one of the key components of cold acclimatization [10,11,12]. However, our results show that there was no significant difference in *DREB1A* and *DREB1B* expression levels in *OsLUX*-overexpressing plants and *oslux* mutants, before or after low-temperature treatment (Figure 6D,E). Promoter analysis showed that the *OsLUX* promoter contains two DREB-binding elements motifs at positions −510/−505 (GTCGGa) and −162/−170 (cCACCGccc), indicating that OsDREB1s and OsDREB2s may regulate *OsLUX* expression by binding to these motifs to respond to cold stress (Figure 7). Thus, OsLUX may act as a downstream gene of the DREB pathway. The transcriptional regulation could be further validated using the available DREB transgenic rice RNA-seq database. However, so far, we have not found a usable RNA-seq database to prove our point. In addition, OsLUX may mediate LUX autoregulation through the LBS (LUX binding site) at position −485/−495 (tccGAATCttg) (Figure 7).

Besides its role in regulating the circadian clock, OsLUX has been shown to be involved in abiotic stress tolerance. Previous studies had demonstrated that loss-of-function mutants of *OsLUX* exhibited lower survival rates under salt stress [35]. In our study, we found that the knockout of *OsLUX* decreased cold tolerance, while the overexpression of *OsLUX* increased cold tolerance at the seedling stage compared to wild-type under cold-stress conditions. Therefore, the *OsLUX* gene in rice plays a critical role in regulating the plant’s circadian rhythm and stress tolerance. By regulating the expression of clock-controlled genes and stress-responsive genes, *OsLUX* allows the plant to synchronize its physiological processes with the day–night cycle and respond to environmental stresses. Further research on *OsLUX* and its involvement in other physiological processes could provide valuable insights into the molecular mechanisms of plant growth and development, as well as strategies for improving crop productivity and stress tolerance [50,51,52].

## 4. Materials and Methods

### 4.1. Plant Material and Treatments

Rice cultivar Zhonghua11 (ZH11, *Oryza sativa* L. subsp. *japonica*) was used in this research article. Seeds were sterilized in 0.3% NaClO and soaked in fresh water at 28 °C for 3 d (with daily water changes). The germinated seeds were sowed on 96-well plastic plates and cultured in Yoshida’s nutrition solution in the growth chamber, with a circadian cycle of 12 h light/28 °C and 12 h dark/22 °C at 500–600 μmol m^−2^s^−1^ light intensity and 60% humidity until the three-leaf stage. In the field experiments, ZH11 wild-type rice and *OsLUX* transgenic rice were grown in natural experiment field under a conventional cultivation environment in the Jiangpu Experiment Station of Nanjing Agricultural University, China.

For the tissue-specific expression profile assay, some three-leaf stage seedlings were transferred to the paddy field until the booting stage, and the rice tissues (including stem, node, leaf blade, leaf sheath, immature panicle, mature panicle, and anther) were harvested at the proper time. 

To explore the expression pattern under cold conditions, the three-leaf-stage seedlings were transferred to a growth chamber with a circadian cycle of 12 h light/4 °C and 12 h dark/4 °C. For ABA treatment, seedlings were transferred to Yoshida’s nutrition solution containing 100 μM ABA. Samples were collected at a series of time points after each treatment, immediately frozen in liquid nitrogen, and were used for further RNA isolation. For diurnal expression analysis, plants were grown for 14 d in the greenhouse under natural day-length conditions of 12 h light/12 h dark, 28 °C, and entrained for 3 d. 

For seedling cold-stress treatment, three-week-old wild-type *OsLUX* and *OsLUX*-overexpression seedlings cultured in Yoshida’s nutrition solution were transferred into a growth chamber under 4 °C conditions for 5 days, with a circadian cycle of 12 h/12 h (light/dark). The survival rate was estimated after recovering for a week, and plants with green leaves were calculated as survivors.

### 4.2. RNA Extraction, cDNA Preparation, and RT-qPCR

Total RNA was extracted using TRIzol reagent (Invitrogen, Waltham, MA, USA) according to the manufacturer’s instructions. Genomic DNA elimination and reverse transcription were performed using the HiScript II Q RT SuperMix for qPCR kit with gDNA wiper (Vazyme, Nanjing, China). Real-time quantitative PCR (RT-qPCR) was performed with the AceQ qPCR SYBR Green Master Mix (Vazyme, China) on the ABI QuantStudio One Real-Time PCR System (Applied Biosystems, Waltham, MA, USA). Reactions contained 10 μL 2× SYBR Green Master Mix reagent, 1 μL of 1:4 diluted reverse-transcription reaction, and 150 nM of each gene-specific primer in a final volume of 20 μL. The following standard thermal profile was used for all PCRs: 50 °C for 2 min; 95 °C for 2 min; 40 cycles of 95 °C for 15 s, 60 °C for 30 s, and 72 °C for 30 s. Relative expression levels were normalized to that of *OsActin1* (LOC_Os03g50885) and presented as 2^−ΔΔCT^. The qPCR primers for the *OsLUX*, *OsAPX1*, *OsAPX2* and *OsPOX1* genes are listed in Table S1.

### 4.3. Subcellular Localization of OsLUX in Onion Epidermal Cells

The coding sequence of *OsLUX* was cloned into SalI/SpeI sites of the pA7-GFP vector to obtain a transcriptional fusion of OsLUX and GFP under the control of the CaMV 35S promoter. The fusion (CaMV35S:OsLUX-GFP) and control (CaMV35S:GFP) plasmids were delivered into onion epidermal cells by particle-bombardment. After bombardment, the bombarded tissues were incubated on 1/2 Murashige and Skoog agar medium in darkness for 24–36 h. The GFP signal was observed with confocal laser scanning microscopy (Nikon ECLIPSE 80i, Tokyo, Japan).

### 4.4. Trans-Activation Analysis of OsLUX

A yeast two-hybrid assay was carried out by using a Clontech system. ORF fragments of OsLUX were inserted into BamHI/EcoRI sites of the bait vector pGBKT7 (pBD) to generate the pBD-OsLUX construct. According to the protocol provided by the manufacturer, pBD-OsLUX, the positive control (pAD-T + pBD-53), and the negative control (empty pBD) were used to transform the yeast strain AH109, containing *HIS3*, *ADE2*, *LacZ* and *MEL1* reporter genes. The transformed strains were streaked onto SD/-Trp or SD/-Trp/-His/-Ade/X-α-gal plates for 3 d.

### 4.5. Genotyping of OsLUX Mutant

The T-DNA insertion of mutant line *OsLUX* with a ZH11 background was obtained from the Rice Mutant Database (RMD), maintained by the National Center of Plant Gene Research (Wuhan) at Huazhong Agricultural University. The genotyping of homozygote seedlings was identified by PCR with gene-specific primers (FP, RP) and a T-DNA border primer (BP). Homozygous mutant plants were then used in semi-quantitative RT-PCR and real-time quantitative RT-PCR assays to test the transcript levels of the corresponding gene normalized to *OsActin1*. The primer sequences are listed in Table S1.

### 4.6. Generation of OsLUX Overexpression Plants

To generate overexpression transgenic rice, the coding sequence of *OsLUX* was amplified and cloned into SacI/KpnI sites linearized by binary expression vector pCAMBIA1300, in which transferred gene expression is under the control of the CaMV 35S promoter. The resulting construct was introduced into *Agrobacterium tumefaciens* EHA105 by the freeze-and-thaw method, and then transformed into the rice cultivar ZH11 using the *Agrobacterium*-mediated method. The T1 transgenic plants were selected by Yoshida’s nutrition solution containing 30 mg/L hygromycin. Seeds from each T1 plant were individually collected. Selected T2 plants were propagated, and homozygous lines of overexpressing plants were confirmed by RT-qPCR analysis.

### 4.7. Measurement of Ion Leakage

Ion leakage was measured using the top leaf of rice seedlings, which were harvested from each line before and under cold-stress treatment. The samples were incubated in 1 mL of distilled water for 60 min, with 60 rpm shaking. The conductivity was determined using a B-173 conductivity meter (Horiba, Kyoto, Japan). Ion leakage was calculated as the ratio of conductivity values between measurements before and after autoclaving.

### 4.8. Statistical Analyses

Statistically significant differences (* *p* <0.05, ** *p* <0.01, *** *p* <0.001) were computed based on Student’s *t*-tests. Data are the means ± standard deviations (SD) of three independent biological replicates.

## Data Availability

Not applicable.

## Supplementary Materials

The following supporting information can be downloaded at: https://www.mdpi.com/article/10.3390/ijms24076727/s1.
