# Supplementary material for: OsLUX Confers Rice Cold Tolerance as a Positive Regulatory Factor
Source: Int J Mol Sci. 2023 Apr 4;24(7):6727. doi: 10.3390/ijms24076727 (PMC10094877; doi:10.3390/ijms24076727)
Supplement: Supplementary file 1 [file ijms-24-06727-s001.zip › ijms-2306509-supplementary.pdf]

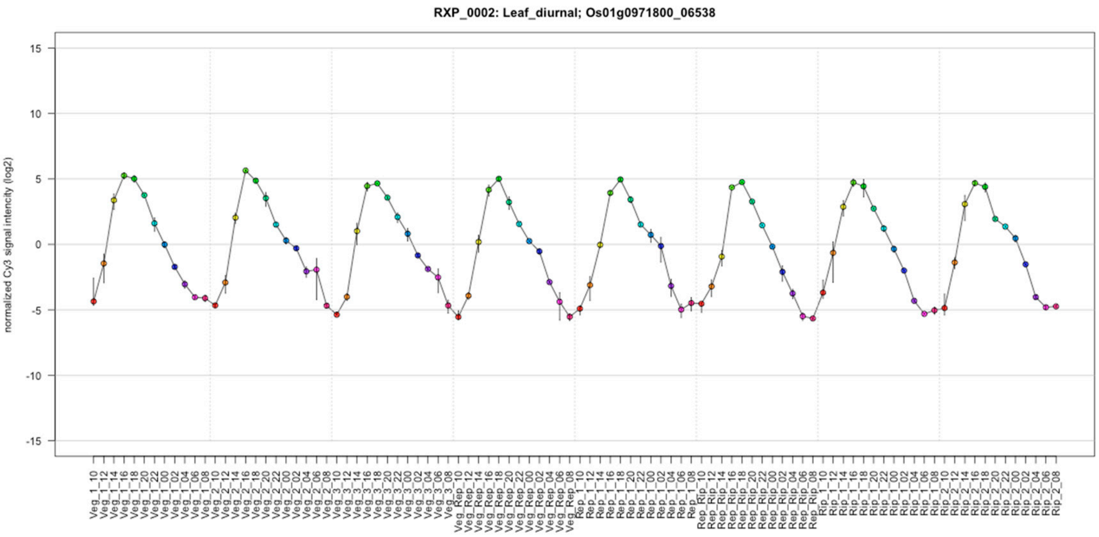

Figure S1. Expression analysis of *OsLUX* response to circadian rhythm in rice leaves under different developmental stages based on RiceXPro microarray data. Veg.: vegetative; Rep.:reproductive; Rip.: ripening.

Table S1. The primer sequences used in this study.

Name	Primer sequences (5'-3')	Purpose
FL-OsLUX-F	AGGAGGAACGAGTGGTTTTTTT	For cloning *OsLUX*
FL-OsLUX-R	GGAGTTTACAGTTGAGGGAGCA	
RT-OsLUX-F	CGAGTATTATGGGCGAGGAG	for *OsLUX* RT-PCR
RT-OsLUX-R	CGGCGTTGTTGTAGGAGTG	
qOsLUX-F	TCCTACAACAACGCCGCCTATG	for *OsLUX* qRT-PCR
qOsLUX-R	GAAGGAATGGATCAGTGGTTGGC	
sub-OsLUX-F	CTCGAGCGAGTATTATGGGCGAG	for *OsLUX* subcellular localization analysis
sub-OsLUX-R	GTCGACGTGGTTGGCGTGGT	
BD-OsLUX-F	GGATCCTTATGGGCGAGGA	for *OsLUX* transcriptional activity analysis
BD-OsLUX-R	GTCGACATCAGTGGTTGGCG	
OE-OsLUX-F	TGAGGTACCATGGGCGAGGA	for *OsLUX* overexpression vector construction
OE-OsLUX-R	ATGTCGACTCAGTGGTTGGCG	
LP	CACGAAACGCACCGTTTAAC	Identification of the *oslux* mutants
RP	GCTCCCTTCGACGCGAAC	
NTLB5	AATCCAGATCCCCCGAATTA	Identification of the *oslux* mutants
PFRB4	TGCAGGTTCTCTCCAAAT	
DBD-OsLUX-F	CCTCTAGATATGGGCGAGGAGG	for *OsLUX* effector plasmids construction
DBD-OsLUX-R	GGTCTAGAGATCAGTGGTTGGCG	
qAPX1-F	AGGTGCCACAAGGAAAGATCTGGT	for *APX1* qRT-PCR
qAPX1-R	TCAGCAGGGCTTTGTCACTAGGAA	
qAPX2-F	TGGGAAGATGCCACAAGGAGAGAT	for *APX2* qRT-PCR
qAPX2-R	TCCGCAGCATATTTCTCCACCAGT	
qPOX1-F	TGATGCTCTGCTTGGTTTCTCC	for *OsPOX1* qRT-PCR
Qpox1-R	GATCTCCTTCGCCTTGGGG	
qOsactin1-F	ATGGTGGTGACGGGTGAC	Internal control for qRT-PCR assay
qOsactin1-R	CAGACACTAAAGCGCCCGGTA	
